# Association between periodontitis and cardiometabolic risk: Results from the Korean National Health and Nutrition Examination Survey 2008-2014

**DOI:** 10.1371/journal.pone.0214731

**Published:** 2019-04-03

**Authors:** Seok Hui Kang, Kyu Hyang Cho, Jun Young Do

**Affiliations:** Division of Nephrology, Department of Internal Medicine, Yeungnam University Hospital, Daegu, Republic of Korea; Sao Paulo State University (UNESP), BRAZIL

## Abstract

**Background:**

Periodontitis and cardiovascular disease (CVD) share inflammation as common pathogenesis. Evaluating the association between periodontitis and CVD would be helpful to better understand the pathophysiology and various complications of periodontitis. We aimed to determine whether there is an independent relationship between periodontitis and various CVD risk indicators or prevalence.

**Patients and methods:**

Our study used representative data from the Korea National Health and Nutrition Examination Survey. Finally, data from 26,097 participants were used for analysis. Periodontitis was defined as a community periodontal index (CPI) ≥3. Participants were classified into 3 groups according to CPI score: Non-PO (participants without periodontitis, CPI score <3), NS-PO (participants with non-severe periodontitis, CPI score = 3), and Severe PO (participants with severe periodontitis, CPI score = 4). Cardiometabolic risk was evaluated based on metabolic syndrome, future CVD risk, and prevalent CVD. Prevalent CVD was defined as participants with cerebrovascular accidents and/or coronary artery disease. Framingham risk score (FRS) was calculated in participants without prevalent CVD.

**Results:**

The numbers of participants in Non-PO, NS-PO, and Severe PO groups were 17,237, 6,738, and 2,122, respectively. The proportions of participants with high FRS and/or prevalent CVD increased as the severity of periodontitis increased. In participants without prevalent CVD, the FRS according to severity of periodontitis increased in both univariate and multivariate analyses as the severity of periodontitis increased. Logistic regression showed that the odds ratio for metabolic syndrome increased as the severity of periodontitis increased on univariate analysis and that the presence of periodontitis was associated with a higher odds ratio for metabolic syndrome on multivariate analysis. Trends for prevalent CVD were similar to those of metabolic syndrome. For participants without prevalent CVD, the odds ratio for high FRS increased as the severity of periodontitis increased in both univariate and multivariate analyses. Subgroup analyses according to sex and age showed similar trends.

**Conclusion:**

Periodontitis was associated with CVD in the Korean population. Therefore, those with periodontitis, especially young adults with severe periodontitis, may be closely monitored for CVD.

## Introduction

Cardiovascular disease (CVD) is a common co-morbidity and the most common cause of death in the general population [1]. Classic risk factors, such as diabetes mellitus, hypertension, age, and life-style, are well-known. Many researchers are working to control these risk factors, but CVD has remained the leading cause of death worldwide. Therefore, research on the identification and control of new risk factors is on-going. The high prevalence of CVD in patients with systemic inflammatory diseases (e.g., rheumatoid arthritis, systemic lupus erythematosus, or chronic kidney disease) demonstrates the association between chronic inflammation and CVD [2–4]. Previous studies have shown that inflammation is associated with metabolic disturbances or vasculopathies, which can lead to CVD [5,6].

Periodontitis is a well-known chronic inflammatory disease with high prevalence rates [7]. Previous studies have shown that patients with periodontitis are prone to dissemination of oral bacteria or endotoxins, which lead to systemic inflammation through an increase in pro-inflammatory cytokines such as tumor necrosis factor-α and interleukin-6 [8]. Recent epidemiologic studies showed that periodontitis contributes significantly to both systemic and localized disease [9]. In addition, many inflammatory cells and mediators are associated with the onset and progression of atherosclerosis through increased production of reactive oxygen species, endothelial dysfunction, and oxidized low-density lipoprotein [10]. Epidemiological data showed the association between inflammation and CVD. Periodontitis and CVD share inflammation as common pathogenesis. Mattila et al. showed a positive association between periodontitis and myocardial infarction [11]. Many subsequent epidemiological studies showed a significant association between periodontitis and CVD, and a recent meta-analysis using 29 cross-sectional studies showed a higher risk of CVD in patients with periodontitis [12]. Some Korean studies investigated the association between periodontitis and metabolic syndrome [13–16]. However, there are few studies regarding the association between periodontitis and risk of future CVD events or prevalence in Korea. We aimed to determine whether there is an independent relationship between periodontitis and various CVD risk indicators or prevalence.

## Patients and methods

### Study population

Our study used representative data from the Korea National Health and Nutrition Examination Survey (KNHANES 2008–2014). The KNHANES is a nationwide, multi-stage, stratified survey of a representative sample of the South Korean population that was conducted by the Korea Centers for Disease Control and Prevention. Restrospective cross-sectional data were collected, including health interviews, health examinations, nutritional surveys, and laboratory investigations [17]. Raw data are available to the public. The total number of participants was 61,379. We excluded participants <30 years old (n = 20,518) or >74 years old (n = 3,887), who could not be evaluated using a modified Framingham risk score (FRS). We also excluded those with insufficient data (n = 10,877). Finally, data from 26,097 participants were used for analysis. Our study was approved by the institutional review board of Yeungnam University Hospital (IRB No: 2018-10-027). The board waived the need for informed consent, as the medical records and information were anonymized and de-identified prior to analysis. Periodontitis was defined as a community periodontal index (CPI) ≥3. Participants were classified into 3 groups according to CPI score: Non-PO (participants without periodontitis, CPI score <3), NS-PO (participants with non-severe periodontitis, CPI score = 3), and Severe PO (participants with severe periodontitis, CPI score = 4).

### Study variables

The following data were collected: age, sex, body mass index (BMI, kg/m^2^), waist circumference (WC, cm), triglyceride levels (TG, mg/dL), high-density lipoprotein cholesterol levels (HDL-C, mg/dL), fasting glucose level (mg/dL), systolic blood pressure (mmHg), diastolic blood pressure (mmHg), smoking habits, physical activity, alcohol intake, presence of periodontitis, and FRS, and history of cerebrovascular accident (CVA), coronary artery disease (CAD), chronic kidney disease (CKD), diabetes mellitus (DM), hypertension (HTN), or metabolic syndrome.

Participant smoking status was classified as non-smoker, ex-smoker, or non-smoker. Physical activity was defined as the presence of regular exercise during leisure time for the past 3 months. Moderate-intensity activities were defined as activities such as job-related activities and light sports such as slow swimming, double tennis, volleyball, badminton, and table tennis. High-intensity activities were defined as activities such as job-related activities and heavy sports such as running, mountain climbing, fast bicycling, fast swimming, soccer, basketball, rope jumping, squash, and single tennis. Physical activity was defined as moderate-intensity activity >30 min/day, for ≥5 days/week, or high-intensity activity >20 min/day, for ≥3 days/week [18]. Alcohol intake was defined according to the Korean definition of standard drinking, based on the World Health Organization (WHO) classification [19]. Participants were classified into 3 groups: abstinence (no alcohol consumption during the 12 months prior to the survey), moderate consumption (women, 0.1–19.99 g of pure alcohol/day; men, 0.1–39.99 g of pure alcohol/day), and heavy consumption (women, ≥20 g of pure alcohol/day; men, ≥40 g of pure alcohol/day).

CVA was defined as a self-reported stroke as diagnosed by a medical doctor. CAD was defined as a self-reported history of myocardial infarction or angina as diagnosed by a medical doctor. The estimated glomerular filtration rate (eGFR) was calculated using the Chronic Kidney Disease Epidemiology Collaboration equation from a previous study [20]. CKD was defined as eGFR < 60 ml/min/1.73m^2^ [21]. DM was defined as fasting glucose level ≥126 mg/dL, self-reported DM as diagnosed by a medical doctor, or the use of anti-diabetic medication [22]. Blood pressure was measured 3 times on the participant’s right arm in a rested and seated position. Final blood pressure was defined as the average of the second and third blood pressure measurements. HTN was defined as blood pressure ≥ 140/90 mmHg, self-reported HTN as diagnosed by as a medical doctor, or the use of anti-hypertensive medication [23].

### Assessment of periodontitis and cardiometabolic risk

The oral examination was performed by trained dentists. The WHO CPI was used to define periodontitis and assess its severity as previously described [7]. Six sextants were examined (posterior right maxilla, anterior maxilla, posterior left maxilla, posterior right mandible, anterior mandible, and posterior left mandible). The CPI was scored from 0 to 4: 0, no bleeding or calculus, and no pocket depth ≥ 4 mm; 1, bleeding on probing but no calculus and no pocket depth ≥ 4 mm; 2, supra- or sub-gingival calculus but no pocket depth ≥ 4 mm); 3, 4 ≤ pocket depth < 6 mm; and 4, pocket depth ≥ 6 mm. All teeth in each sextant were evaluated, and the highest score was defined as the CPI for each participant.

Cardiometabolic risk was evaluated based on metabolic syndrome, future CVD risk, and prevalent CVD. Metabolic syndrome was defined according to the Adult Treatment Panel III criteria using the modified cutoff values for Asian populations as suggested by the Asia-Pacific guidelines [24]. Prevalent CVD was defined as participants with CVA and/or CAD. FRS was calculated in participants without prevalent CVD. FRS is a well-known scoring system for prediction of 10-year CVD risk. Wilson et al. developed the original version of the FRS in 1998. The version was greatly useful for predicting coronary heart disease, including angina pectoris, myocardial infarction, coronary insufficiency, and coronary artery disease-related death. However, the original version did not include cerebrovascular events as primary outcomes. [25]. Therefore, D’Agostino et al. developed the modified Framingham risk score system using coronary artery disease and cerebrovascular events as primary outcomes. The modified scoring system was useful to predict cardiovascular risk, including coronary artery disease and cerebrovascular events [26]. We calculated the risk scores for each subject based on this system. The FRS groups were defined by risk percentages, i.e., low risk (<10%), intermediate risk (10–20%), and high risk (>20%).

### Statistical analysis

The data were analyzed using statistical software SPSS (Version 23.0; IBM Corp., Armonk, NY, USA). Categorical variables were expressed as both counts and percentages. Continuous variables were expressed as the mean ± standard error. Pearson’s χ^2^ or Fisher’s exact test were used to analyze categorical variables. Continuous variables were analyzed using one-way analysis of variance, followed by a post-hoc Tukey comparison for univariate analysis and analysis of covariance for multivariate analysis. Logistic regression analyses were used to estimate the odds ratios (OR) and 95% confidence intervals (CI), which were then used to determine the relationship between periodontitis and metabolic syndrome, high FRS, or prevalent CVD. Multivariate analyses were adjusted for age, sex, smoking habit, alcohol intake, DM, HTN, physical activity, eGFR, and metabolic syndrome. A *P*-value < 0.05 was considered statistically significant.

## Results

### Clinical characteristics of participants

The numbers of participants in Non-PO, NS-PO, and Severe PO groups were 17,237, 6,738, and 2,122, respectively (Table 1). Participants in the Non-PO group were younger than those in the other groups. There were more females than in the Non-PO group than in the other groups. BMI, WC, HDL-C, fasting blood glucose, and systolic blood pressure increased as the severity of periodontitis increased. The proportion of current smoking and heavy alcohol intake increased as the severity of periodontitis increased. The prevalence of CVA, CAD, CKD, DM, and HTN increased as the severity of periodontitis increased.

**Table 1:** Clinical characteristics of the participants according to periodontitis severity.

Variables	Non-PO (n = 17,237)	NS-PO (n = 6,738)	Severe PO (n = 2,122)	*P*-value^a^
Age (years)	48.4 ± 0.1	57.6 ± 0.1^b^	56.1 ± 0.2 ^b^^c^	<0.001
Sex (men, %)	6432 (37.3%)	3467 (51.5%)	1291 (60.8%)	<0.001
Body mass index (kg/m^2^)	23.72 ± 0.03	24.22 ± 0.04^b^	24.44 ± 0.07 ^b^^c^	<0.001
Waist circumference (cm)	80.7 ± 0.1	83.6 ± 0.1 ^b^	84.5 ± 0.2 ^b^^c^	<0.001
Triglycerides (mg/dL)	131.4 ± 0.8	151.5 ± 1.5 ^b^	156.4 ± 2.6^b^	<0.001
HDL-C (mg/dL)	52.7 ± 0.1	50.4 ± 0.1 ^b^	49.0 ± 0.3 ^b^^c^	<0.001
Fasting blood glucose (mg/dL)	97.1 ± 0.2	102.2 ± 0.3 ^b^	106.7 ± 0.7 ^b^^c^	<0.001
Systolic blood pressure (mmHg)	117.2 ± 0.1	122.3 ± 0.2 ^b^	123.5 ± 0.4 ^b^^c^	<0.001
Diastolic blood pressure (mmHg)	76.3 ± 0.1	78.2 ± 0.1 ^b^	78.6 ± 0.4^b^	<0.001
Participants with high physical activity (%)	8691 (50.4%)	3572 (53.0%)	1099 (51.8%)	<0.001
Smoking habits within each group				0.001
Participants with non-smoking habit	11292 (65.5%)	3459 (51.3%)	910 (42.9%)	
Participants with ex-smoking habit	1811 (10.5%)	752 (11.2%)	335 (15.8%)	
Participants with current smoking habit	4134 (24.0%)	2527 (37.5%)	877 (41.3%)	
Cerebrovascular accident (%)	235 (1.4%)	159 (2.4%)	75 (3.5%)	<0.001
Coronary artery disease (%)	323 (1.9%)	192 (2.8%)	82 (3.9%)	<0.001
Chronic kidney disease (%)	315 (1.8%)	207 (3.1%)	71 (3.3%)	<0.001
Alcohol intake				<0.001
Abstinence	4706 (27.3%)	2019 (30.0%)	553 (26.1%)	
Moderate intake	11807 (68.5%)	4329 (64.2%)	1418 (66.8%)	
Heavy intake	724 (4.2%)	390 (5.8%)	151 (7.1%)	
Participants with prevalent diabetes mellitus (%)	1524 (8.8%)	1043 (15.5%)	467 (22.0%)	<0.001
Participants with prevalent hypertension (%)	4394 (25.5%)	2531 (37.6%)	887 (41.8%)	<0.001

Data are expressed as numbers (percentages) for categorical variables and as the mean ± standard error for continuous variables. Abbreviations: Non-PO, participants without periodontitis; NS-PO, participants with non-severe periodontitis; Severe PO, participants with severe periodontitis; HDL-C, high-density lipoprotein cholesterol.

^a^*P-*values were tested using the one-way analysis of variance, followed by a post-hoc Tukey comparison, for continuous variables and Pearson’s χ^2^ or Fisher’s exact test for categorical variables.

^b^*P* <0.05 compared with the Non-PO.

^c^*P* <0.05 compared with the NS-PO.

### Relationship between the severity of periodontitis and cardio-metabolic risk

The numbers of participants with low FRS, intermediate FRS, high FRS, and prevalent CVD were 12,218 (70.9%), 2,390 (13.9%), 2,087 (12.1%), and 542 (3.1%) in the Non-PO group, 3,283 (48.7%), 1,498 (22.2%), 1,627 (24.1%), and 330 (4.9%) in the NS-PO group, and 763 (36.0%), 540 (25.4%), 667 (31.4%), and 152 (7.2%) in the Severe PO group, respectively (*P* <0.001). The proportions of participants with high FRS and/or prevalent CVD increased as the severity of periodontitis increased.

**Fig 1:**
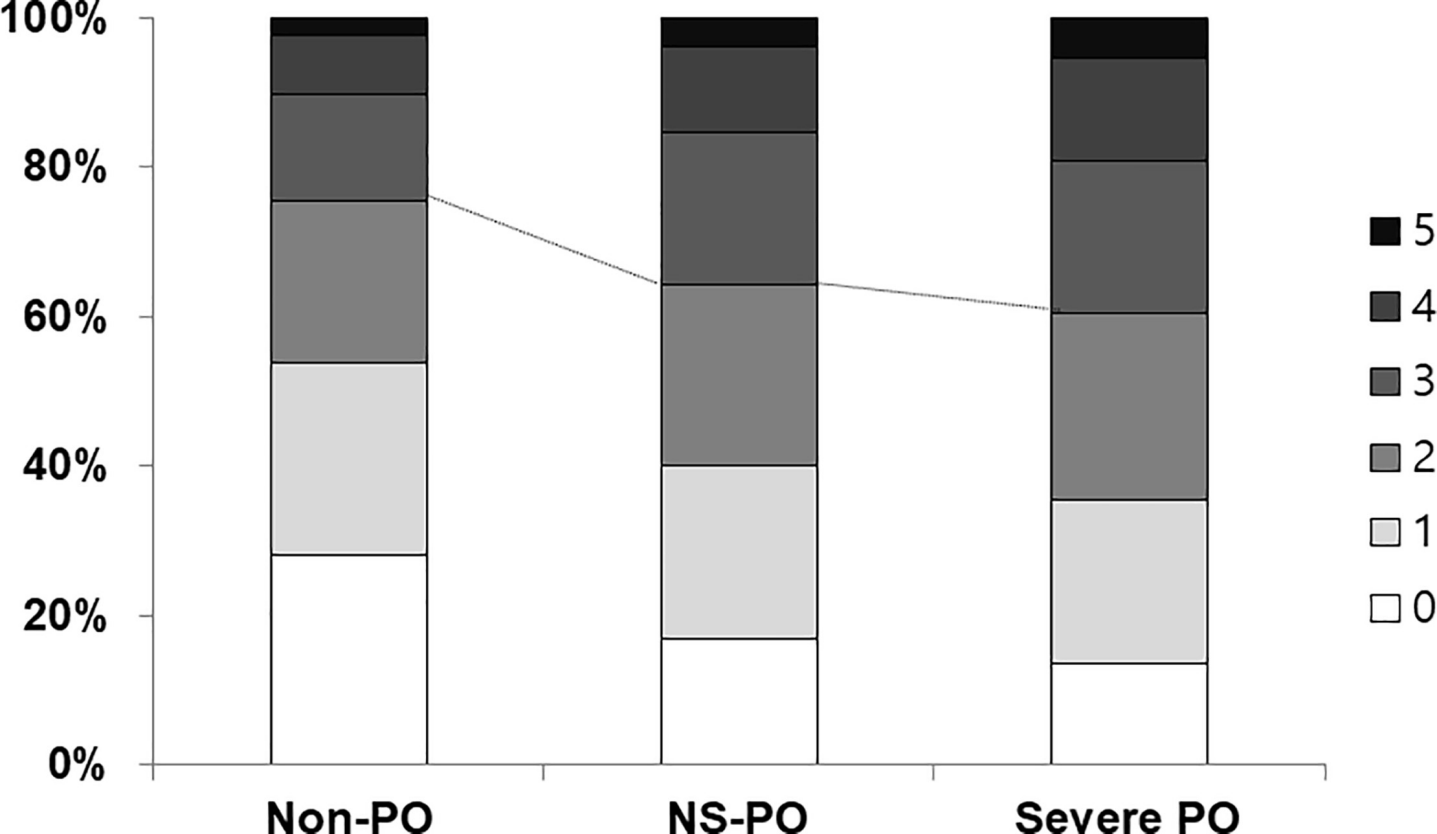
Number of metabolic syndrome components according to periodontitis severity. The numbers of participants with 0, 1, 2, 3, 4, or 5 metabolic syndrome components were 4,818 (28.0%), 4,442 (25.8%), 3,733 (21.7%), 2,439 (14.2%), 1,378 (8.0%), and 389 (2.3%) in the Non-PO group; 1,130 (16.8%), 1,559 (23.2%), 1,629 (24.2%), 1,367 (20.3%), 778 (11.6%), and 264 (3.9%) in the NS-PO group; and 291 (13.7%), 461 (21.7%), 532 (25.1%), 433 (20.4%), 290 (13.7%), and 113 (5.4%) in the Severe PO group, respectively (*P* < 0.001). Abbreviation: Non-PO, participants without periodontitis; NS-PO, participants with non-severe periodontitis; Severe PO, participants with severe periodontitis.

**Fig 2:**
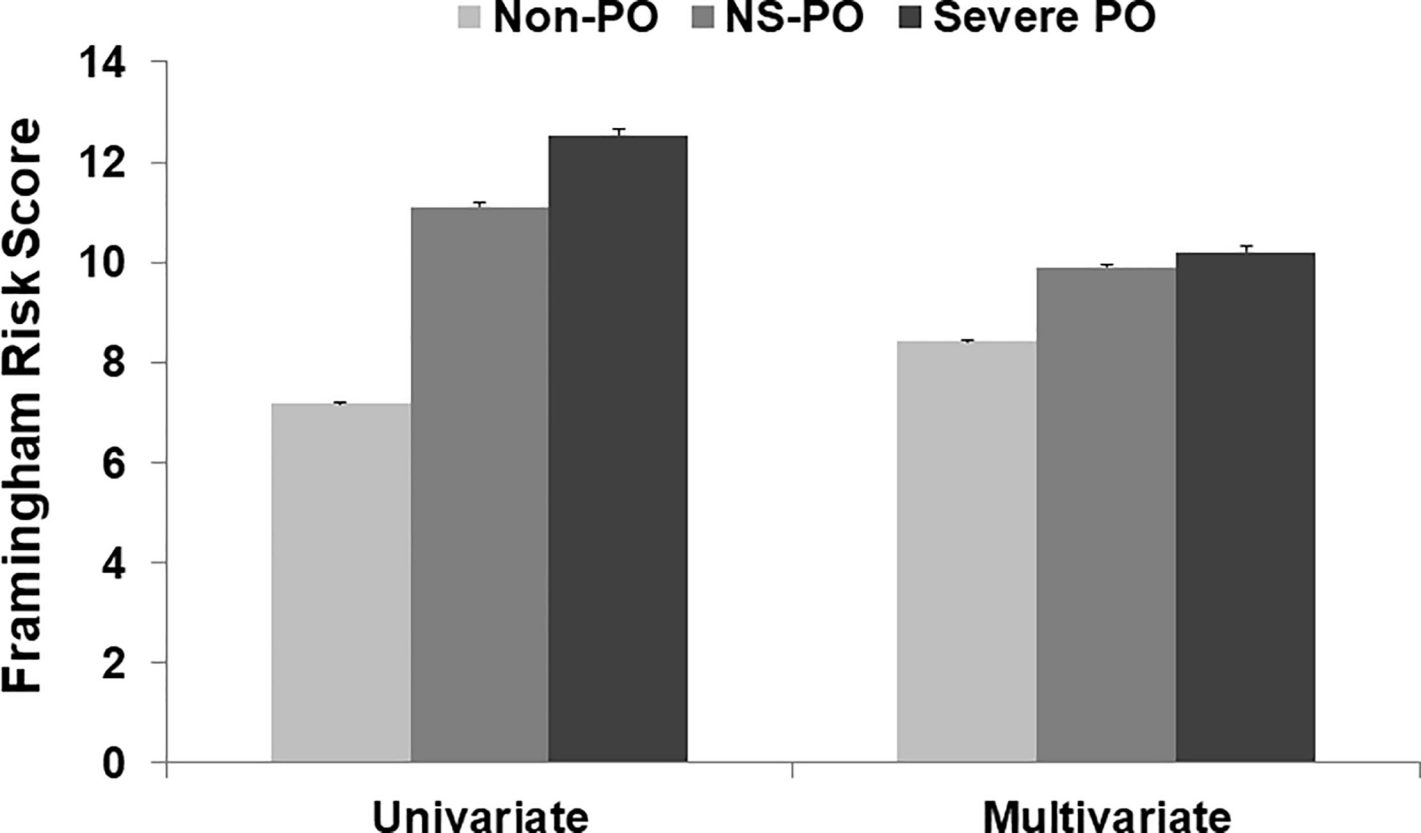
Framingham risk score according to periodontitis severity. The data are shown as mean and standard error values. In univariate analysis, the Framingham risk score in the Non-PO, NS-PO, and Severe PO groups was 7.17 ± 0.05, 11.11 ± 0.08, and 12.54 ± 0.13, respectively (*P* < 0.001). In multivariate analysis, the risk score in the Non-PO, NS-PO, and Severe PO groups was 8.42 ± 0.03, 9.91 ± 0.05, and 10.21 ± 0.10, respectively (*P* < 0.001). The multivariate analysis was adjusted for age, sex, smoking habits, alcohol intake, diabetes mellitus, hypertension, physical activity, estimated glomerular filtration rate, and the presence of metabolic syndrome. Abbreviations: Non-PO, participants without periodontitis; NS-PO, participants with non-severe periodontitis; Severe PO, participants with severe periodontitis.

The numbers of participants with metabolic syndrome were 4,214 (24.4%) in the Non-PO group, 2,413 (35.8%) in the NS-PO group, and 837 (39.4%) in the Severe PO group, respectively (*P* <0.001). The proportions of participants with 2 or more metabolic syndrome components also increased as the severity of periodontitis increased (Fig 1). In participants without prevalent CVD, the FRS according to severity of periodontitis increased in both univariate and multivariate analyses as the severity of periodontitis increased (Fig 2).

Logistic regression showed that the odds ratio (OR) for metabolic syndrome increased as the severity of periodontitis increased on univariate analysis and that the presence of periodontitis was associated with a higher OR for metabolic syndrome on multivariate analysis (Table 2). Trends for prevalent CVD were similar to those of metabolic syndrome. For participants without prevalent CVD, the OR for high FRS increased as the severity of periodontitis increased in both univariate and multivariate analyses.

**Table 2:** Logistic regression analyses of cardiometabolic risks according to periodontitis severity.

	Metabolic syndrome		High FRS		Prevalent CVD	
	OR (95% CI)	*P*-value*	OR (95% CI)	*P*-value^#^	OR (95% CI)	*P*-value^#^
**Univariate**						
Non-PO (reference)						
NS-PO	1.72 (1.62–1.83)	<0.001	2.38 (2.21–2.56)	<0.001	1.61 (1.40–1.85)	<0.001
Severe PO	2.01 (1.83–2.21)	<0.001	3.58 (3.23–3.98)	<0.001	2.38 (1.97–2.86)	<0.001
NS-PO (reference)						
Severe PO	1.17 (1.06–1.29)	0.002	1.51 (1.35–1.68)	<0.001	1.48 (1.21–1.80)	<0.001
**Multivariate**						
Non-PO (reference)						
NS-PO	1.32 (1.23–1.42)	<0.001	1.33 (1.19–1.48)	<0.001	1.10 (0.95–1.28)	0.197
Severe PO	1.31 (1.17–1.46)	<0.001	1.72 (1.48–2.01)	<0.001	1.50 (1.23–1.82)	<0.001
NS-PO (reference)						
Severe PO	1.03 (0.92–1.16)	0.589	1.29 (1.10–1.52)	0.002	1.35 (1.10–1.66)	0.004

*The multivariate analysis was adjusted for age, sex, smoking habits, alcohol intake, diabetes mellitus, hypertension, physical activity, and estimated glomerular filtration rate.

^**#**^The multivariate analysis was adjusted for age, sex, smoking habits, alcohol intake, diabetes mellitus, hypertension, physical activity, estimated glomerular filtration rate, and metabolic syndrome.

Abbreviation: OR, odds ratio; CI, confidence interval; FRS, Framingham risk score; CVD, cardiovascular disease; Non-PO, participants without periodontitis; NS-PO, participants with non-severe periodontitis; Severe PO, participants with severe periodontitis.

### Subgroup analyses by sex and age

**Fig 3:**
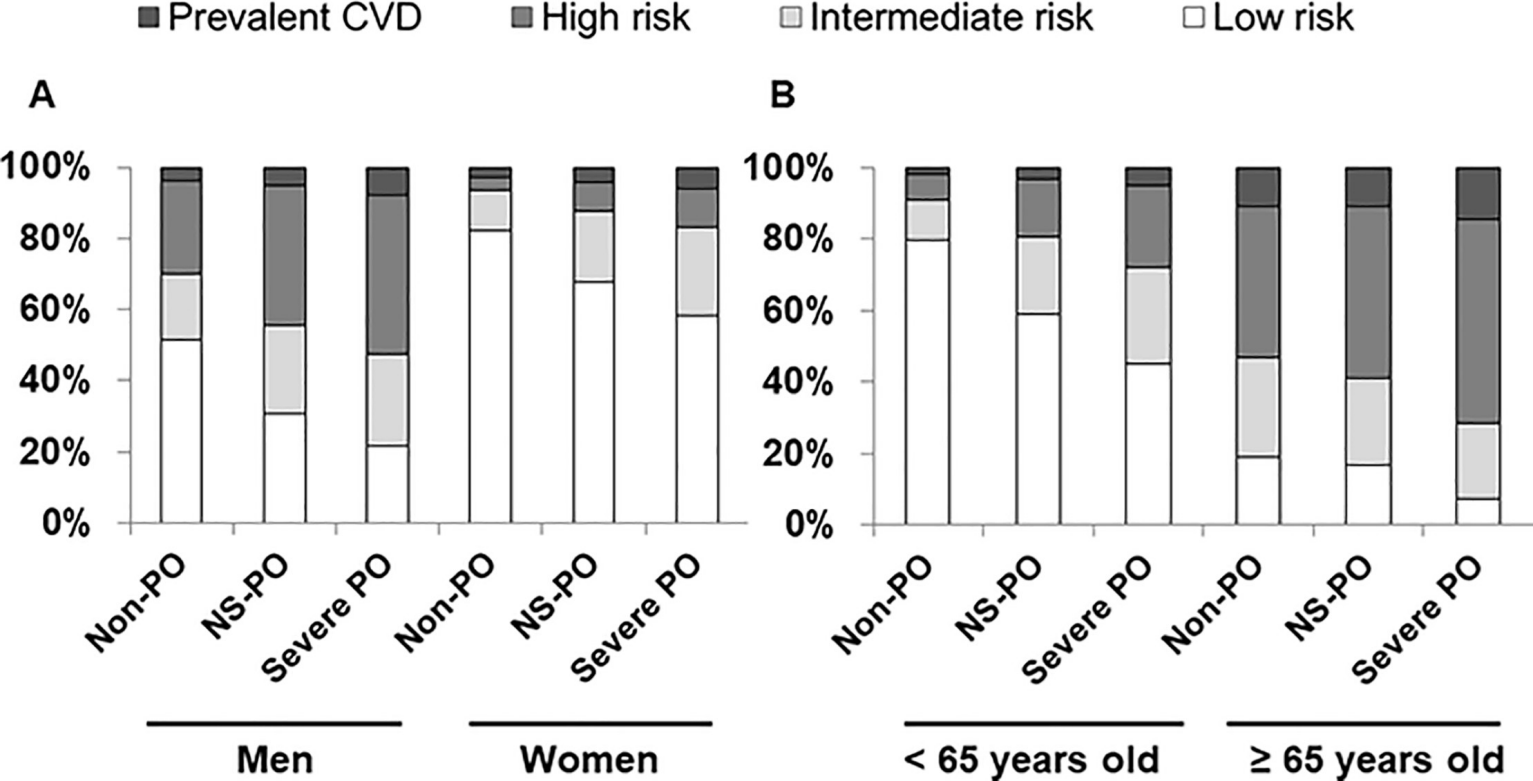
Subgroup analyses according to sex and age. **Fig A:** Among men, the numbers of participants with low FRS, intermediate FRS, high FRS, and prevalent CVD were 3,325 (51.7%), 1,187 (18.5%), 1,676 (26.1%), and 244 (3.7%) in the Non-PO group; 1,070 (30.9%), 851 (24.5%), 1,363 (39.3%), and 183 (5.3%) in the NS-PO group; and 278 (21.5%), 335 (25.9%), 578 (44.8%), and 100 (7.8%) in the Severe PO group, respectively (*P* < 0.001). Among women, the numbers of participants with low FRS, intermediate FRS, high FRS, and prevalent CVD were 8,893 (82.3%), 1,203 (11.1%), 411 (3.8%), and 298 (2.8%) in the Non-PO group; 2,213 (67.7%), 647 (19.8%), 264 (8.1%), and 147 (4.4%) in the NS-PO group; and 485 (58.3%), 205 (24.7%), 89 (10.7%), and 52 (6.3%) in the Severe PO group, respectively (*P* < 0.001). **Fig B:** Among participants < 65 years old, the numbers with low FRS, intermediate FRS, high FRS, and prevalent CVD were 11,741 (79.6%), 1,696 (11.5%), 1,038 (7.0%), and 276 (1.9%) in the Non-PO group; 3,007 (58.9%), 1,103 (21.6%), 839 (16.4%), and 156 (3.1%) in the NS-PO group; and 724 (45.2%), 431 (27.0%), 368 (23.0%), and 76 (4.8%) in the Severe PO group, respectively (*P* < 0.001). Among participants ≥ 65 years old, the numbers with low FRS, intermediate FRS, high FRS, and prevalent CVD were 477 (19.2%), 694 (27.9%), 1,049 (42.2%), and 266 (10.7%) in the Non-PO group; 276 (16.9%), 395 (24.2%), 788 (48.3%), and 174 (10.6%) in the NS-PO group; and 39 (7.5%), 109 (20.8%), 299 (57.2%), and 76 (14.5%) in the Severe PO group, respectively (*P* <0.001). Abbreviations: FRS, Framingham risk score; CVD, cardiovascular disease; Non-PO, participants without periodontitis; NS-PO, participants with non-severe periodontitis; Severe PO, participants with severe periodontitis.

The numbers of men and women participants were 11,190 and 14,907, respectively. Those <65 years old and ≥65 years old numbered 21,455 and 4,642, respectively. Among men, the numbers of participants with metabolic syndrome in Non-PO, NS-PO, and Severe PO groups were 1,696 (26.4%), 1,140 (32.9%), and 439 (34.0%). Among women, the numbers of participants with metabolic syndrome Non-PO, NS-PO, and Severe PO groups were 2,518 (23.3%), 1,273 (38.9%), and 398 (47.9%). Among young participants (< 65 years old), the numbers of participants with metabolic syndrome in Non-PO, NS-PO, and Severe PO groups were 3,116 (21.1%), 1,638 (32.1%), and 581 (36.3%). Among elderly participants (≥65 years old), the numbers of participants with metabolic syndrome in Non-PO, NS-PO, and Severe PO groups were 1,098 (44.2%), 775 (47.5%), and 256 (48.9%) (*P* < 0.001 for men, women, <65 years old and *P* = 0.038). The proportions of participants with metabolic syndrome, high FRS, or prevalent CVD increased as the severity of periodontitis increased (Fig 3).

## Discussion

The proportion of participants with periodontitis was approximately 34.0%. The proportions of participants with metabolic syndrome, high FRS, or prevalent CVD increased as the severity of periodontitis increased. Logistic regression analysis showed that metabolic syndrome was associated with the presence of periodontitis, high FRS was associated with the severity of periodontitis, and prevalent CVD was associated with severe periodontitis alone. Subgroup analyses according to sex and age showed similar trends.

Periodontitis is one of most common health problems in the world [27]. Chronic diseases such as DM are associated with a high prevalence of periodontitis [7]. Periodontitis also leads to the development of chronic diseases. It is an inflammatory disease that involves the gingiva, cementum, periodontal ligament, and alveolar bone. Infection or tissue injuries by periodontitis can lead to systemic inflammation through increases in the levels of various inflammatory cytokines/mediators. Inflammation is associated with CVD via various pathways such as the development of coronary atherosclerosis [10]. Therefore, the relationship between periodontitis and CVD may be associated with chronic inflammation. However, the association can be influenced by some confounding factors such as age, sex, physical activity, BMI, dyslipidemia, smoking habit, alcoholic intake, DM, and blood pressure. We performed multivariate and subgroup analyses using possible confounding factors to decrease these effects. Univariate analyses revealed a positive association between the two variables. In addition, multivariate and subgroup analyses showed similar results.

Previous clinical studies have shown that periodontitis is positively associated with metabolic syndrome through mechanisms such as low-grade inflammation, and lipid or vascular abnormalities [28–30]. A meta-analysis of 20 studies showed a positive association between periodontitis and metabolic syndrome [31]. Studies of ethnic or national differences are ongoing. Some studies have investigated the association between periodontitis and metabolic syndrome in the Korean population, and 5 studies showed a positive association [13–16,32]. Among 5 studies, 4 enrolled participants in a limited region within Korea or only small numbers of participants [13,15,16,32]. One study used a representative sample from KNHANES but analyzed data collected at 1 year [14]. Our study used a representative sample from KNHANES 2008–2014 and enrolled the largest sample. The proportion with metabolic syndrome and the numbers of metabolic syndrome components increased as the severity of periodontitis increased. Logistic regression analysis showed that the presence of periodontitis was associated with a high OR compared with the absence of periodontitis, but statistical significance in a comparison between NS-PO and Severe PO groups was not evident. Statistically significant differences in elderly participants with metabolic syndrome were observed among the 3 groups, but the association was weak.

Dietrich et al. analyzed 12 cohort studies and showed that periodontitis is associated with incident CVD and that the positive association was stronger in younger than in older individuals [33]. The joint European Federation of Periodontology/American Academy of Periodontology workshop based on this meta-analysis found a consistent and strong association between the 2 variables [34]. A recent study of a nationwide cohort showed an association between periodontitis and CVD mortality beyond incident CVD [35]. Research continues to show a definite association. In Korea, previous studies investigated the association between periodontitis and subclinical atherosclerosis, peripheral artery disease, hypertension, and stroke, but these were cross-sectional studies and evaluated prevalent outcomes [36–41]. A cohort study design using follow-up data is more appropriate for identification of a causal-relationship, but such a study requires major expense and effort. A predictive model or scoring system using cross-sectional data may be useful for overcoming these problems. The FRS system is a well-known and validated method for prediction of future CVD risk in participants without prevalent CVD. We analyzed the cross-sectional data and evaluated the association between periodontitis and prevalent CVD or future CVD risk using the FRS. Our data revealed that both the presence and severity of periodontitis were associated with high FRS in participants without prevalent CVD. Prevalent CVD was associated with severe periodontitis alone.

This study had several limitations. First, this was a retrospective, cross-sectional study. We could not evaluate a causal-association between periodontitis and cardiometabolic risk. Second, we defined CVD as a self-reported diagnosis, as given by a medical doctor. Our study did not include data for more precise methods such as coronary angiography, brain imaging, or echocardiography. Subclinical CVD might have been overlooked. Third, our data did not include inflammatory markers. The purpose of the study was to evaluate the association between periodontitis and CVD. The relationship may be associated with inflammation. Data for inflammatory markers would be useful to identify the association between the two variables. However, this study was evaluated using KNHANES data. The database did not include data for inflammation. Therefore, our study did not include the data. In addition, the database did not include postprandial blood glucose, random glucose, and HbA1c levels. Therefore, DM was defined on the basis of fasting blood glucose and a history of DM. A prospective study that included accurate parameters using imaging methods and inflammatory markers, is warranted to overcome these limitations.

In conclusion, periodontitis was associated with CVD in the Korean population. Therefore, those with periodontitis, especially young adults with severe periodontitis, may be closely monitored for CVD.
